# Trends in the use of dental prostheses among adults and the elderly and the effects of their provision in the Brazilian health system 2003–2023

**DOI:** 10.1590/1980-549720260011.supl.1

**Published:** 2026-07-20

**Authors:** Sonia Cristina Lima Chaves, Thais Regis Aranha Rossi, Jony Arrais Pinto, Thamyres Pereira Lima, Ana Tereza Ribeiro da Silva, Ana Maria Freire de Souza Lima, Andréia Cristina Leal Figueiredo, Joana Danielle Brandão Carneiro, Leonardo Marchini, Monira Samann Kallás, Doralice Severo da Cruz Teixeira

**Affiliations:** I Universidade Federal da Bahia, Institute of Public Health – Salvador (BA), Brazil.; II Universidade do Estado da Bahia – Salvador (BA), Brazil.; III Universidade Federal Fluminense – Niterói (RJ), Brazil.; IV Secretaria de Saúde do Estado da Bahia – Salvador (BA), Brazil.; V Universidade Federal da Bahia – Salvador (BA), Brazil.; VI Universidade Federal da Bahia, Faculty of Dentistry – Salvador (BA), Brazil.; VII Ministério da Saúde – Brasília (DF), Brazil.; VIII The University of Iowa College of Dentistry and Dental Clinics – Iowa City, Iowa, United States of America.; IX Hospital Sírio-Libanês – São Paulo (SP), Brazil.

**Keywords:** Dental prosthesis, Oral health services, Health policy, Health impact assessment

## Abstract

**Objective:**

This study analyzed trends in the use of dental prostheses among adults and older adults and their availability in the Unified Health System (SUS) in Brazil between 2003 and 2023.

**Methods:**

Quasi-experimental ecological observational study for impact evaluation using a *Difference-in-Differences* (DiD) model. The temporal variation was compared between municipalities that implemented Dental Prosthesis Laboratories (LPD) (intervention) and those that did not (counterfactual). Data from 43 municipalities participating in all SB Brasil (Brazil Oral Health) epidemiological surveys from 2003, 2010, and 2023 were used. The outcome variables were the increased use of complete dentures (CD), removable partial dentures (RPD), and fixed dentures in adults (35–44 years old) and older adults (65–74 years old) in these municipalities.

**Results:**

The presence of LPD between 2003 and 2010 was associated with a 311.96% increase in the supply of prostheses (p=0.032), but it remained stable between 2010 and 2023. Changes in government probably impacted the supply of prostheses in these municipalities. The presence of LPD in the period 2003–2010 and having an Dental Specialty Center (CEO) between 2010 and 2023 increased the use of CD among adults by 6.94% (p=0.012) and 10.5% (p=0.032). Among older adults, having LPD increased the use of RPD by 12.42% (p=0.002), and being in a municipality with a CEO increased the use of CD by 12.05% (p=0.05) between 2003 and 2010.

**Conclusion:**

The LPD supply had a residual effect. The lower tooth loss may explain the reduced use of dentures in adults and the increased use of RPD in older adults. Future analyses are suggested regarding coverage, access, and different contexts.

## INTRODUCTION

Tooth loss remains a global public health problem, with significant impacts on people's quality of life^1^, although findings from SB Brasil 2023 indicate a decline in Brazil among adults and older adults^2^. The consequences go beyond impairment of oral and masticatory functions, extending to psychological and social aspects, making tooth loss a challenge for health systems^3,4^.

The Brazilian state's social response was the formulation of a policy to provide dental prostheses within the Unified Health System (SUS) in 2004, through the strategy of Regional Dental Prosthesis Laboratories, referred to in this study as LPD, which carry out the laboratory stage of the prosthesis production, coordinated with its clinical stage in primary or specialized health care services^5^. In 2024, Brazil delivered about 1,237,151 dental prostheses through the SUS^6^, the majority (51.5%) of which were complete dentures (CD).

Access to dental prostheses through the public health system, in the model adopted by Brazil, is uncommon in others countries^7–9^. When it does occur, these services are often purchased from the private sector, at costs to individuals^8,10^, which are identified as an obstacle to population access to these oral health services^9,10^.

Studies that have evaluated this component of the National Oral Health Policy (PNSB) point to difficulties in the supply of specialists and in the training of teams; in the planning and programming of activities; in assessing prosthetic needs in the population; and in monitoring and analyzing oral health indicators; in addition to low funding^11,12^, lack of organizational support from managers^11,13^ and absence of specialized dental services^12,13^. An increase in supply has been observed, but with inequality in its distribution^14–16^, longer waiting times, and the need to resort to the private sector to obtain dental prostheses^17^.

There is a scarcity of research with an evaluative focus on the impact of public policies, and its incorporation has been recommended^18^. It was hypothesized that the expansion of the supply of dental prostheses in the SUS would increase their use among adults and older adults in the municipalities that benefited, compared to those that did not. Thus, this study evaluated the trends in use and the effect of federal induction by the LPD on the supply of dental prostheses in the SUS from 2003 to 2023 among Brazilian adults and older adults.

## METHODS

Ecological quasi-experimental observational study assessing impact using a *Difference-in-Differences* (DiD) model^18^, a before-and-after design with a counterfactual group, based on the SB Brasil epidemiological surveys from 2003, 2010, and 2023, including only municipalities that participated in all three surveys (n=43). The sample size was determined by the availability of data from the national oral health surveys conducted in the period analyzed, resulting in the inclusion of 43 municipalities with complete and comparable information over time.

The analysis was conducted in two stages. In the first stage, descriptive comparisons of indicators stratified by groups with and without LPD were performed, in some cases involving a small number of municipalities and asymmetric distributions. For this reason, it was decided to consistently use non-parametric tests in all comparisons between the beginning and the end of the period, with results presented as medians and interquartile ranges. In the second stage, a linear regression model was fitted followed by a residual analysis.

Initially, two distinct time windows (2003–2010 and 2010–2023) were analyzed, considered independently. In each of them, the percentages of use of CD, removable partial dentures (RPD), and fixed prostheses (FP) were compared between the beginning and the end of the period, stratified by municipalities with and without LPD and by two age groups (35–44 and 65–74 years). The rate of prosthesis provision per 100,000 inhabitants in the selected municipalities was also compared between the beginning and the end of each period, stratified by municipalities with and without LPD. In all scenarios, the indicators were described using the median and interquartile range, and comparisons were performed using the Wilcoxon test.

The two times windows were also analyzed from the perspective of different government periods, considered in this study as a specific segment of the conjuncture, delimited by the time interval during which a governing team is in power and responsible for the direction and implementation of public policies^19^.

For the impact analysis, the DiD^18^ model was used as a strategy to estimate the effect of the implementation of LPDs. This method compares the temporal variation in the outcome between two groups: municipalities with LPD implementation (intervention) and municipalities without LPD (control). The outcome considered was the percentage use of CD, RPD, and FP before and after the policy, in each time window (2003–2010 and 2010–2023), analyzed separately for the two age groups (35–44 and 65–74 years). The estimated linear model was:


$$y_{it}=\beta_{0}+\beta_{1}LPD_{it}+\beta_{2}Ano_{it}+\beta_{3}LPD:Ano_{it}+\gamma x_{it}+\varepsilon_{it},$$

in which:

*y_it_* = average proportion of prosthesis use (total, removable or fixed) in municipality i in year t;

*LPD_it_* = indicator that takes the value 1 if municipality i has implemented LPD in year t, and 0 otherwise;

*Ano_it_* = indicator that takes the value 1 for the final year of each window, and 0 for the initial year;

*x_it_* = Vector of control variables, including capital status, presence of a CEO, and variation in the illiteracy rate between the years evaluated (lower values of this indicator suggest an improvement in the municipality).

*ΰ*_0_ = intercept of the model;

*ΰ*_1_ = Captures average differences between municipalities with and without LPD;

*ΰ*_2_ = average effect of the time between the two considered periods;

*ΰ*_3_ = coefficient that estimates the DiD effect of LPD implementation;

*γ* = Vector of parameters for the control variables;

*ε_it_* = error term.

The normality of the residuals was assessed using the Shapiro-Wilk test. The significance level adopted for the analyses was 5%, all performed using R software (version 4.4.3)^20^.

The outcome variables (percentage of dental prosthesis use) were constructed based on aggregated percentages recorded by municipality in each age group in 2003, 2010, and 2023, based on the individuals examined. The average percentage of individuals using some type of prosthesis in the upper and lower arches in each age group was calculated for each municipality in the three surveys. It is important to note that the data from the SB Brasil 2003, 2010, and 2023 surveys result from complex and independent sampling designs. In the present study, the outcomes were operationalized as aggregated proportions by municipality. It is recognized that it would be possible to calculate weighted proportions according to individual weights. However, since individual sample weights are not available for SB Brasil 2003, an ecological and comparative approach was chosen, without individual weighting.

To determine the type of dental prosthesis produced, data was collected from the SUS Outpatient Information System (SIA/SUS) on the number of prostheses produced by registered dental prostheses in the 43 municipalities included in this study, from January to December of each year. The annual rate of dental prosthesis procedures per 100,000 inhabitants was calculated by dividing the total number of dental prostheses produced in each registered municipality by the number of the municipality's population over 14 years of age, multiplied by 100,000. For this purpose, the population figures were obtained using data from the studies "Study of population estimates by municipality, sex and age – 2000-2021" and "Projection of the Population of the federative units by sex, simple age and age groups: 2010-2060 (2018 edition)", available at: https://datasus.saude.gov.br/informacoes-de-saude-tabnet/^21^.

The nomenclature and codes for outpatient procedures related to dental prostheses in the Brazilian Unified Health System (SUS) were changed in 2008. Therefore, for procedures performed up to 2007, information was collected on the following procedures: 1008210 (mandibular complete denture), 1008211 (maxillary complete denture), 1008213 (mandibular complete denture), 1008214 (maxillary complete denture), 1008302 (removable maxillary/mandibular partial dentures), 1008303 (mandibular removable partial denture), 1008304 (maxillary removable partial denture), 1008406 (fixed partial denture per element), 1008407 (metal-ceramic adhesive prosthesis per element), 1008408 (metal-plastic adhesive prosthesis per element). From 2008 onwards, information was collected on outpatient procedures: complete mandibular prosthesis (SIGTAP code 07.01.07.012-9), complete maxillary prosthesis (SIGTAP code 07.01.07.013-7), removable partial mandibular prosthesis (SIGTAP code 07.01.07.009-9), removable partial maxillary prosthesis (SIGTAP code 07.01.07.010-2) and fixed/adhesive coronary/intraradicular retainer (per element) (SIGTAP code 07.01.07.014-5)^6^.

Among the 43 municipalities analyzed, 31 implemented the LPD, with most municipalities with LPD starting their implementation in 2009 (n=16, 37.2%) or between 2010 and 2014 (n=11, 25.6%). The national databases used are products of the SB Brasil 2003, 2010, and 2023 surveys, and their results are currently a publicly available database with free access. The study report followed the recommendations of the STROBE checklist (*Strengthening the Reporting of Observational Studies in Epidemiology*). The table is available as Supplementary Material 1.

### Data availability statement:

The entire dataset supporting the results of this study are available within the article.

## RESULTS

In the period 2003–2010, there was a statistically significant increase in the supply of dental prostheses in municipalities with LPD. The median rose from 0.4 to 306.3 prostheses per 100,000 inhabitants (p=0.0074). In municipalities without LPD, there was no significant change. Between 2010 and 2023, no difference in supply was observed in either group (Table 1). In the DiD analysis, the presence of LPD in 2003–2010 was associated with a 311.96% increase in prosthesis supply (p=0.032) (Table 2).

**Table 1:** Median and lower and upper quartiles of the outcome variables "dental prosthesis provision in the SUS" (total population) and "use of dental prostheses by type" (total, removable partial, or single fixed) among adults and older adults in the years 2003–2010 and 2010–2023, p-value and trend in municipalities with and without LPD in Brazil (n=43).

SB Brazil period and presence of LPD and outcome variable		2003–2010			2010–2023		
		2003	2010	p-value and trend	2010	2023	p-value and trend
		median (IIQ)	median (IIQ)		median (IIQ)	median (IIQ)	
Rate per 100,000 for prosthesis supply							
	Without LPD	0	0.3	0.0884	0	0	0.2733
		(0–37.4)	(0–92.7)	(Stable)	(0–9.7)	(0–5.3)	(Stable)
	With LPD	0.4	306.3	**0.0074**	93.3	80	0.1447
		(0–36.7)	(53.1–410.9)	(Increase)	(0.3–376.2)	(11.0–175.5)	(Stable)
% of use of CD among 35–44-year-olds							
	Without LPD	18.2	7.8	**0.0014**	12.1	4.3	0.1441
		(13.3–24.1)	(5.3–16.0)	(Decrease)	(7.7–23.1)	(1.9–11.8)	(Stable)
	With LPD	21.1	7.9	**0.0019**	7.7	2.2	**0.0012**
		(14.7–27.5)	(5.9–8.9)	(Decrease)	(5.5–8.7)	(0.9–3.5)	(Decrease)
% usage of RPD among ages 35–44 years							
	Without LPD	30.2	23.1	**0.0345**	24.5	15.5	0.1614
		(20.3–33.3)	(18.1–28.8)	(Decrease)	(21.6–28.5)	(9.1–23.2)	(Stable)
	With LPD	22.6	16.3	0.2343	19.4	7.5	**<0.0001**
		(21.5–28.8)	(12.4–33.1)	(Stable)	(14.9–31.8)	(4.9–12.9)	(Decrease)
% of FP use among ages 35–44 years							
	Without LPD	10.3	3.8	**0.0413**	4.1	4	1.0000
		(5.1–12.3)	(1.1–7.3)	(Decrease)	(1.2–8.0)	(3–4.6)	(Stable)
	With LPD	7	2.9	**0,0341**	3	2.2	0.5663
		(3,5 - 11,6)	(1–4.2)	(Decrease)	(1.1–6.4)	(1.1–3.5)	(Stable)
% of CD usage among 65–74-year-olds							
	Without LPD	55.2	59.6	0.8291	61.5	62.5	0.9528
		(50–69.0)	(54.5–63.4)	(Stable)	(57.3–64.1)	(43.0–76.90	(Stable)
	With LPD	60	61.1	0.554	59.2	51	**0.0014**
		(51.7–64.3)	(55.6–65.3)	(Stable)	(54.5–64.6)	(46.7–59.4)	(Stable)
% of RPD usage among 65–74-year-olds							
	Without LPD	14.3	2.6	0.1208	12.5	25	**0.0180**
		(9.1–16.7)	(0.9–12.8)	(Stable)	(5.7–13.6)	(18.4–28.6)	(Increase)
	With LPD	21.4	2.4	**0.0231**	2.4	23.9	**0.0026**
		(17.4–28.6)	(1.2–6.7)	(Decrease)	(1.2–5.5)	(20.0–26.7)	(Increase)
% of use of FP at ages 65–74 years							
	Without LPD	10.4	9.5	0.8785	10.5	2	0.1088
		(6.8–15.4)	(6.2–13.1)	(Stable)	(6.5–12.5)	(1.7–2.70)	(Stable)
	With LPD	10	7.5	0.7532	7.8	4.8	**<0.0001**
		(5.3–12.3)	(4.8–12.6)	(Stable)	5.3–13.1	(3.7–7.3)	(Decrease)

The bold p-values confirm the statistical significance of the association adopted for the analyses, which was 5% (<0.05).

LPD: Dental Prosthesis Laboratories. RPD: Removable Partial Dentures. CD: Complete Dentures. FP: Fixed Prosthesis.

Source: Data from SB Brasil 2003, 2010, and 2023.

**Table 2:** Effect of the presence of LPD on the variation in supply (total population) and the use of dental prostheses among adults (35–44 years) in the periods 2003–2010 and 2010–2023 in selected municipalities (n=43) based on the DiD model

Variable	Estimated effect	std.error	statistic	p-value	Variable	Estimated effect	std.error	statistic	p-value
2003–2010					2010–2023				
Prosthesis supply rate per 100,000 inhabitants.					Prosthesis supply rate per 100,000 inhabitants.				
Intercept	51,691	160,466	0,322	0,748	Intercept	255,359	526,804	0,485	0,630
Illiteracy Rate 2010–2003	-1,259	11,108	-0,113	0,910	Illiteracy Rate 2023–2010	6,963	23,543	0,296	0,769
CEO 2003–2010 (Yes)	-12,391	133,023	-0,093	0,926	CEO (Yes)	-73,057	475,186	-0,154	0,878
Capital	-2,939	73,605	-0,040	0,968	Capital	**-236,645**	**111,782**	**-2,117**	**0,039**
Year (2010)	41,954	131,249	0,320	0,750	Year (2023)	-142,809	445,413	-0,321	0,750
LPD (Yes)	74,735	101,738	0,735	0,465	LPD (Yes)	305,243	522,931	0,584	0,562
Year (2010): LPD (Yes)	**311,960**	**143,016**	**2,181**	**0,032**	Year (2023): LPD (Yes)	-72,367	456,170	-0,159	0,875
**Use of fixed prosthesis**					**Use of fixed prosthesis**				
Intercept	14,501	4,820	3,008	0,004	Intercept	10,009	1,749	5,724	< 0,001
Illiteracy Rate 2010–2003	**-0,546**	**0,208**	**-2,626**	**0,011**	Illiteracy Rate 2023–2010	0,120	0,128	0,936	0,354
CEO 2003–2010 (Yes)	-2,273	4,754	-0,478	0,634	CEO (Yes)	-3,464	2,363	-1,466	0,149
Capital	**-2,730**	**1,268**	**-2,153**	**0,035**	Capital	**-5,219**	**1,004**	**-5,199**	**<0,001**
Year (2010)	-6,246	4,715	-1,325	0,190	Year (2023)	-1,556	2,009	-0,774	0,442
LPD (Yes)	-2,595	1,608	-1,614	0,112	LPD (Yes)	-1,226	1,634	-0,750	0,457
Year (2010): LPD (Yes)	0,340	2,293	0,148	0,883	Year (2023): LPD (Yes)	0,183	2,165	0,084	0,933
**Use of removable partial dentures**					**Use of removable partial dentures**				
Intercept	19,658	5,341	3,680	<0,001	Intercept	26,508	4,506	5,883	<0,001
Illiteracy Rate 2010–2003	-0,117	0,374	-0,313	0,755	Illiteracy Rate 2023-2010	-0,004	0,315	-0,013	0,990
CEO 2003–2010 (Yes)	6,412	4,421	1,450	0,151	CEO (Yes)	2,523	4,772	0,529	0,599
Capital	1,566	2,505	0,625	0,534	Capital	**-10,316**	**2,894**	**-3,565**	**0,001**
Year (2010)	1,841	4,309	0,427	0,670	Year (2023)	-5,100	4,875	-1,046	0,299
LPD (Yes)	-2,173	3,342	-0,650	0,518	LPD (Yes)	4,233	4,546	0,931	0,355
Year (2010): LPD (Yes)	2,043	4,791	0,426	0,671	Year (2023): LPD (Yes)	-5,892	5,503	-1,071	0,288
**Use of complete dentures**					**Use of complete dentures**				
Intercept	17,653	4,473	3,946	<0,001	Intercept	15,028	3,813	3,942	<0,001
Illiteracy Rate 2010–2003	0,219	0,292	0,751	0,455	Illiteracy Rate 2023-2010	0,449	0,313	1,437	0,156
CEO 2003–2010 (Yes)	5,834	3,799	1,536	0,129	CEO (Yes)	**10,500**	**4,774**	**2,199**	**0,032**
Capital	**-9,670**	**2,077**	**-4,655**	**<0,001**	Capital	**-12,816**	**2,951**	**-4,343**	**<0,010**
Year (2010)	-1,668	3,772	-0,442	0,660	Year (2023)	1,727	4,796	0,360	0,720
LPD (Yes)	**6,936**	**2,682**	**2,586**	**0,012**	LPD (Yes)	5,201	3,848	1,352	0,182
Year (2010): LPD (Yes)	-6,132	3,905	-1,570	0,121	Year (2023): LPD (Yes)	-4,522	5,229	-0,865	0,391

The p-values in bold confirm the statistical significance of the association adopted for the analyses, which was 5% (<0.05).

Source: Data from SB Brasil 2003, 2010 and 2023.

**Figure 1:**
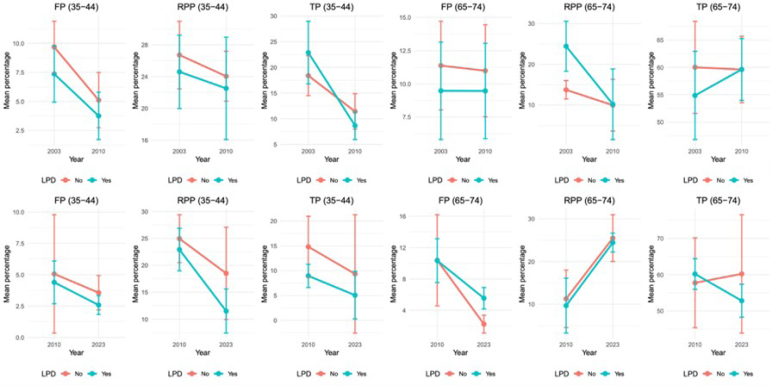
Mean percentage and 95% confidence interval for the use of dental prostheses, according to type of prosthesis and age group (35–44 and 65–74 years), in municipalities with and without participation in the Dental Prosthesis Laboratory Policy. The first line shows the comparison for the period 2003–2010 and the second, for the period 2010–2023.

Among adults aged 35–44 years, the use of prostheses decreased significantly or remained stable across all types between 2003 and 2010, in both municipalities with and without LPD. In municipalities without LPD, the reduction was significant between 2003 and 2010, dropping from 30.2% to 23.1% (p=0.0345). For FP, there was a reduction in both groups with and without LPD (p=0.04 and p=0.03, respectively). For TP, the median fell from 18.2% to 7.8% (p=0.0014) in municipalities without LPD and from 21.1% to 7.9% (p=0.0019) in those with LPD (Table 1). Already in the 2010–2023 period, among adults the reduction was again significant for CD and RPD in the group with LPD, reaching only 2.2% use of CD among adults in 2023 and showing stability in the groups of municipalities without LPD (Table 1, Figure 1).

In the DiD analysis for the 2003–2010 period, it is noteworthy that the reduction in the illiteracy rate was associated with lower use of fixed prostheses in adults by -0.546% (p=0.011). Being in a state capital reduced the use of FP and DP by -2.73% (p=0.035) and -9.67% (<0.001), respectively. Additionally, municipalities with LPD showed an increase in the use of DP of 6.94% (p=0.012). In the 2010–2023 period, living in a state capital was associated with a reduction in the use of all types of prostheses among adults. On the other hand, the use of DP among adults increased by 10.5% (p=0.032) in municipalities with CEO (Table 2).

Among older adults aged 65 to 74 years, in the period 2003–2010, there was stability in almost all types, except for a decrease in the use of RPD in municipalities with LPD (p=0.0231). In turn, in the period 2010–2023, the significant increase in the use of RPD in municipalities with and without LPD stands out. For the use of CD, there was stability in municipalities with (p=0.0014) and without LPD (p=0.9528), with no significance. For the period 2003–2010, the estimated effect was a 4.5% increase in the use of CD in municipalities with LPD, but it was not significant (Table 3). Additionally, among the elderly in capital cities there was an association with a greater reduction in the use of RPD of 13.5% (p<0.001) between 2003 and 2010, and a reduction of 15.65% (p<0.001) between 2010 and 2023. On the other hand, between 2003 and 2010, in municipalities with LPD, there was an association with a greater use of RPD of 12.42% (p=0.002). In the period 2010–2023, in municipalities with CEO, there was an association with a greater use of CD in this group of 12.05% (p=0.049).

**Table 3:** Effect of the presence of LPD on the variation in the use of dental prostheses among elderly people (65–74 years) in the periods 2003–2010 and 2010–2023 in selected municipalities (n=43) based on the DiD model.

Variable	Estimated effect	std.error	statistic	p-value	Variable	Estimated effect	std.error	statistic	p-value
2003–2010					2010–2023				
% of Use of fixed prosthesis					Use of fixed prosthesis				
Intercept	10,814	5,458	1,981	0,054	Intercept	13,153	4,221	3,116	0,003
Illiteracy Rate 2010–2003	-0,036	0,324	-0,110	0,913	Illiteracy Rate 2023–2010	0,454	0,271	1,673	0,100
CEO (Yes)	1,086	4,576	0,237	0,813	CEO (Yes)	3,714	5,274	0,704	0,484
Capital	-0,953	2,491	-0,383	0,704	Capital	**-6,195**	**2,020**	**-3,066**	**0,003**
Year (2010)	0,801	4,411	0,182	0,857	Year (2023)	-4,388	4,781	-0,918	0,363
LPD (Yes)	-1,634	3,382	-0,483	0,631	LPD (Yes)	3,608	3,925	0,919	0,362
Year (2010): LPD (Yes)	0,361	4,195	0,086	0,932	Year (2023): LPD (Yes)	-0,997	5,020	-0,199	0,843
**Percentage variation of use of removable partial dentures**					**Use of removable partial dentures**				
Intercept	18,534	6,354	2,917	0,005	Intercept	18,873	3,979	4,743	<0,001
Illiteracy Rate 2010–2003	-0,483	0,445	-1,085	0,283	Illiteracy Rate 2023–2010	0,095	0,255	0,374	0,710
CEO 2003–2010 (Yes)	4,333	5,368	0,807	0,423	CEO (Yes)	-6,195	3,978	-1,557	0,124
Capital	**-13,506**	**3,009**	**-4,488**	**< 0,001**	Capital	**-15,650**	**2,566**	**-6,100**	**<0,001**
Year (2010)	-0,461	5,386	-0,086	0,932	Year (2023)	**14,987**	**4,365**	**3,433**	**<0,001**
LPD (Yes)	**12,426**	**3,865**	**3,215**	**0,002**	LPD (Yes)	3,425	4,126	0,830	0,409
Year (2010): LPD (Yes)	-9,798	5,365	-1,826	0,073	Year (2023): LPD (Yes)	-1,193	4,966	-0,240	0,811
**Percentage variation in the use of complete dentures**					**Use of complete dentures**				
Intercept	65,163	8,938	7,290	< 0,001	Intercept	55,057	6,306	8,730	<0,001
Illiteracy Rate 2010–2003	-0,547	0,619	-0,884	0,380	Illiteracy Rate 2023–2010	0,542	0,409	1,324	0,189
CEO 2003–2010 (Yes)	-3,016	7,409	-0,407	0,685	CEO (Yes)	**12,046**	**6,040**	**1,994**	**0,049**
Capital	-1,607	4,100	-0,392	0,696	Capital	-6,849	3,735	-1,834	0,071
Year (2010)	-2,607	7,311	-0,357	0,722	Year (2023)	4,215	6,717	0,628	0,532
LPD (Yes)	-6,184	5,667	-1,091	0,278	LPD (Yes)	10,350	6,278	1,649	0,103
Year (2010): LPD (Yes)	4,525	7,966	0,568	0,572	Year (2023): LPD (Yes)	-11,593	7,604	-1,525	0,131

The bold p-values confirm the statistical significance of the association adopted for the analyses, which was 5% (<0.05).

Source: Data from SB Brasil 2003, 2010, and 2023.

Regarding trends, what stands out is the decrease or stability in the use of prostheses among adults. Among the elderly, there was also stability or a decrease in the percentage of prosthesis use, with the exception of an increase in the use of removable partial dentures (RPD) in the second period in this group in municipalities with and without LPD (Table 1).

## DISCUSSION

The findings of the present study indicate that the rate of prosthesis provision per 100,000 inhabitants increased between 2003 and 2010, but remained stable between 2010 and 2023. The contextual changes, which were more favorable in the first period – the Lula I and II administrations (2003–2010) – and more restrictive at the end of the second period (2010–2023), probably impacted the provision of prostheses in the municipalities analyzed. The PNSB, also known as Brasil Sorridente (Smiling Brazil), was one of the four priority policies on the federal agenda from 2003 onward, and the public provision of dental prostheses began to receive regular federal support with the creation of the LPD^16,22^. Paim^23^ notes that political and social forces began to occupy space during this period, with strengthening of social policies on the political agenda.

There was an increase in federal financial transfers for oral health through 2013 and maintenance of values between 2013 and 2016; however, there was a reduction starting in 2017 and a sharp drop in 2018^24^. This aligns with the increase in the rate of prosthesis provision per 100,000 inhabitants in the first period, but the instabilities in governments after 2015 reduced the federal government's capacity for policy induction, combined with difficulties at the local level. The financial crisis experienced by Brazil in 2008 did not have direct effects on oral health funding, due to the rapid recovery by the federal government. However, between 2011 and 2016, the economic and political scenario faced austerity measures; thus, the space for consolidating a national project for the expansion of social policies was restricted^24^.

The period from 2016–2022 was marked by the deepening of fiscal austerity measures at the national level, as well as counter-reforms and political crises in the Brazilian State^25,26^. The policy of providing dental prostheses expanded the installed capacity of the SUS, although this expansion did not translate proportionally into greater use by the population, as reflected in the higher proportion of prosthesis use. It is worth noting that the implementation of Dental Prosthesis Laboratories (LPD) in municipalities was concentrated between 2009 and 2010, with substantial growth in LPD over the period and the highest recorded number in 2023 (n=3,737) – an increase of approximately 42 times compared to 2005, with most of them being privately owned in legal terms^6,21^. In addition, a previous study revealed that, in municipalities with LPD, the median increased from 36.7 prostheses/100,000 inhabitants in 2003 to 376.2 in 2010 (p=0.031)^27^. These studies suggest that the implementation of LPD acted as a driver of prosthesis production within the SUS, enabling greater availability of this type of rehabilitation^27^.

The unobserved impact on the greater use of dental prostheses in municipalities with LPD requires careful analysis. First, the DiD analysis highlights that the 20-year period between the three surveys exerted a significant influence on the reduction in prosthesis use among adults, albeit to a lesser extent among the elderly^2,3^. Furthermore, although the number of municipalities with dental prosthesis services increased during this period, the supply of prostheses showed variations below the funding ranges induced by the Ministry of Health^6^. The reasons may be related to difficulties in bidding processes, with discontinuation of contracts with private dental prosthesis providers and a low number of private laboratories interested in participating in tenders. Finally, municipalities with larger populations, such as capital cities, which make up the majority of the present sample, have a low concentration of dental prostheses per inhabitant^15,21^. In summary, the sample in this study includes all capital cities, which, even with dental prosthesis services, may have low coverage resulting in low access and, therefore, may not impact the prosthesis use indicator.

Regarding competitive bidding models, these can result in complex negotiations, extended deadlines, increased costs, and a drop in service quality, suggesting the need for rigorous monitoring^28^.

A study indicated that municipalities with a greater presence of CEO (Dental Specialty Center) and ESB (Oral Health Team) had a higher supply^29^, but even so, the number of prostheses delivered was below expectations, and the trend remained stable. Furthermore, the study also revealed that racial inequalities persist, since, even where there is a greater supply, the black population receives proportionally fewer prostheses than the white population, with a difference of 7.7% to the detriment of blacks. This inequality is more pronounced in cities with more ESB and/or a higher proportion of black population^29^.

National studies indicate that the need for dental prostheses among elderly Brazilians is high, but has been decreasing over the last few surveys^2,3,29,30^, consequently leading to a lower prevalence of prosthesis use^29,30^. However, it is worth highlighting that this need is especially greater in low-income populations, non-white skin color, and lower education levels^29,30^. Individuals with lower education and income have a greater need for and less use of prostheses, reflecting historical inequalities in access to oral health^4,30,31^. Longitudinal studies show stability in the prevalence of prosthesis use among the elderly in São Paulo between 2000 and 2010, without a significant reduction during the period^32^. In the case of epidemiological surveys, a reduction in the need for prostheses was observed among adults from 75.9% in 2010 to 53.57% in 2023. Among the elderly, 96% needed prostheses of any type in 2010, and in 2023, this percentage decreased to 75.2%^2,3^.

The decrease in the use of prostheses is a consequence of changes in the oral health profile of the population, with a reduction in tooth loss in some age groups due to the expansion of prevention and oral health promotion policies^33^. The increase in the use of removable partial dentures (RPDs) is probably also a product of the lower tooth loss observed in the historical series in Brazil. In European countries, about half of adults have some type of dental prosthesis, with a trend towards a reduction in edentulism and an increase in fixed prostheses and implants^34^. In this sense, the incorporation of fixed and implant-supported prostheses can be an important resource for oral rehabilitation, already consolidated in other countries^34^, with the advancement of digital technologies^34^.

Despite the widespread adoption of standardized instruments such as the *Oral Health-Related Quality of Life* (OHRQoL)^35^, no studies were found using robust impact assessment methods, such as DiD or Interrupted Time Series (ITS), for public policies or oral health programs. Systematic and scoping reviews indicate that most assessments focus on clinical or self-reported outcomes, focusing on school groups or children, without exploring causal inference methods for large-scale policies^36,37^. In this sense, the present study proposes outcomes related to the stated objective of the policy and suggests incorporating specific measures based on theoretical models. The adoption of impact assessment methods such as DiD and ITS is recommended to strengthen the evidence on the effectiveness of oral health policies, especially in broader contexts, using already available information systems^36,19^.

This study has limitations related to the use of secondary data, aggregated by municipality. Furthermore, the data from the SB Brasil 2003, 2010, and 2023 surveys result from complex and independent sampling designs. However, in this study, the outcomes were operationalized as proportions aggregated by municipality, and the analytical objective was to estimate relative temporal variations and differences between groups of municipalities, not to produce national population estimates. It is recognized that it would be possible to calculate weighted proportions based on individual weights. However, sample weights are not available for SB Brasil 2003, which would make their consistent incorporation throughout the analyzed historical series unfeasible. Therefore, an ecological and comparative approach was chosen, recognizing this decision as a methodological limitation of the study. Another limitation was that the final sample only included municipalities present in all 3 surveys (n=43), which resulted in a greater representation of capital cities and large municipalities. To mitigate possible regional imbalances, we included an indicator variable "being a capital city" in the adjusted models.

It is suggested that impact assessment studies incorporate individualized data focusing on social position (income and education), type of service used, need for prostheses, and perceptions of oral health. It is also recommended that new studies investigate differences in prosthesis provision by population size and organizational network, as well as analyses of the period 2003–2023. Considering the national scope of the data and the universal nature of the Brazilian Unified Health System (SUS), the findings have the potential for generalization to similar contexts.

Finally, tooth loss, understood as an expression of social inequities and poor access to dental care throughout life, still constitutes an important public health problem that underpinned the dental prosthesis policy in the SUS^5,31^.
