# Supplementary material for: Trends in the use of dental prostheses among adults and the elderly and the effects of their provision in the Brazilian health system 2003–2023
Source: Rev Bras Epidemiol. 2026 Jul 20;29(Suppl 1):e260011supl1. doi: 10.1590/1980-549720260011.supl.1 (PMC13403701; doi:10.1590/1980-549720260011.supl.1)
Supplement: Supplementary Material 1 [file 1980-5497-rbepid-29-suppl1-e260011supl1-supp01.docx]

**Material Suplementar 1.** Checklist STROBE para Estudos Observacionais.

| **Item** | **Recomendação STROBE** | **Local** | **Ajustes realizados** |
| --- | --- | --- | --- |
| 1 | Indicar o delineamento no título/resumo | Título e Resumo | Trata-se de um estudo observacional ecológico, retrospectivo, de avaliação de impacto, com abordagem quase-experimental por meio do método de diferenças-em-diferenças |
| 2 | Contexto científico e justificativa | Introdução | Adequado |
| 3 | Objetivos e hipóteses | Introdução (final) | Explicitar hipótese. Atendido. O objetivo foi avaliar o impacto da ampliação da oferta de próteses dentárias no SUS sobre indicadores populacionais de uso de prótese dentária. Partiu-se da hipótese de aumento do uso e redução de desigualdades ao longo do tempo. |
| 4 | Delineamento do estudo | Métodos (início) | Concentrar descrição no primeiro parágrafo. Atendido. Estudo observacional ecológico, retrospectivo, com abordagem quase-experimental, baseado na comparação temporal pré e pós-implementação da política, utilizando diferenças-em-diferenças. |
| 5 | Contexto (setting) | Métodos | Adequado |
| 6 | Participantes/unidades | Métodos | Detalhar critérios de inclusão/exclusão. Atendido ao final do primeiro parágrafo. As unidades de análise foram municípios com dados comparáveis nos inquéritos nacionais de saúde bucal. Foram excluídos aqueles que não participaram de todos os inquéritos. |
| 7 | Variáveis | Métodos | Adequado |
| 8 | Fontes de dados | Métodos | Adequado |
| 9 | Viés | Discussão | Mencionar também no Métodos. Atendido. Considerou-se a possibilidade de vieses decorrentes do uso de dados secundários e agregados e de diferenças metodológicas entre inquéritos |
| 10 | Tamanho do estudo | Métodos | Justificar n=43. Atendido. O tamanho amostral foi definido pela disponibilidade de dados completos e comparáveis, resultando na inclusão de 43 municípios. |
| 11 | Variáveis quantitativas | Métodos | Adequado |
| 12 | Métodos estatísticos | Métodos | Sugerir inclusão de IC95%. Atendido, as análises incluíram testes estatísticos apropriados e modelos de diferenças-em-diferenças, com estimativas apresentadas com intervalos de confiança de 95%. |
| 13 | Participantes (fluxo) | Resultados | Após aplicação dos critérios de elegibilidade, 43 municípios compuseram a análise final |
| 14 | Dados descritivos | Resultados | Incluir tabela descritiva. |
| 15 | Dados de desfecho | Resultados | Adequado |
| 16 | Resultados principais | Resultados | Os resultados dos desfechos principais são apresentados em tabelas, com estimativas e medidas de efeito. Os efeitos estimados pelo modelo de diferenças-em-diferenças foram apresentados com intervalos de confiança de 95%. |
| 17 | Outras análises | Resultados | Não foram realizadas análises adicionais além daquelas previamente definidas |
| 18 | Resultados-chave | Discussão | Adequado. Os principais achados são sintetizados no início da discussão, à luz dos objetivos do estudo. |
| 19 | Limitações | Discussão | Adequado. As limitações do delineamento ecológico e do uso de dados secundários são discutidas de forma explícita. |
| 20 | Interpretação | Discussão | Adequado. Os resultados são interpretados considerando o contexto das políticas públicas de saúde bucal no Brasil. |
| 21 | Generalização | Discussão | Considerando a abrangência nacional dos dados e o caráter universal do SUS, os achados apresentam potencial de generalização para contextos semelhantes. |
| 22 | Financiamento | Informações finais | O financiamento não teve influência no delineamento do estudo, na análise dos dados, na interpretação dos resultados ou na decisão de publicação |

**Fonte:** Artigo *“Tendências no uso de prótese dentária entre adultos e idosos e efeitos da sua oferta no Sistema de Saúde brasileiro entre 2003 e 2023”*
