# Supplementary material for: Trends in the use of dental prostheses among adults and the elderly and the effects of their provision in the Brazilian health system 2003–2023
Source: Rev Bras Epidemiol. 2026 Jul 20;29(Suppl 1):e260011supl1. doi: 10.1590/1980-549720260011.supl.1 (PMC13403701; doi:10.1590/1980-549720260011.supl.1)
Supplement: Translation

# Translation: Tendências no uso de prótese dentária entre adultos e idosos e efeitos da sua oferta no sistema de saúde brasileiro entre 2003 e 2023

**DOI:** 10.1590/1980-549720260011.supl.1.2

**Authors:** Sonia Cristina Lima Chaves, Thais Regis Aranha Rossi, Jony Arrais Pinto, Thamyres Pereira Lima, Ana Tereza Ribeiro da Silva, Ana Maria Freire de Souza Lima, Andréia Cristina Leal Figueiredo, Joana Danielle Brandão Carneiro, Leonardo Marchini, Monira Samann Kallás, Doralice Severo da Cruz Teixeira

**Affiliations:** I Universidade Federal da Bahia, Instituto de Saúde Coletiva – Salvador (BA), Brasil.; II Universidade do Estado da Bahia – Salvador (BA), Brasil.; III Universidade Federal Fluminense – Rio de Janeiro (RJ), Brasil.; IV Secretaria de Saúde do Estado da Bahia – Salvador (BA), Brasil.; V Universidade Federal da Bahia – Salvador (BA), Brasil.; VI Universidade Federal da Bahia, Faculdade de Odontologia – Salvador (BA), Brasil.; VII Ministério da Saúde – Brasília (DF), Brasil.; VIII The University of Iowa College of Dentistry and Dental Clinics – Iowa City, Iowa, United States of America.; IX Hospital Sírio-Libanês – São Paulo (SP), Brasil.

**Keywords:** Prótese dentária, Serviços de saúde bucal, Política de saúde, Avaliação do impacto na saúde

## Abstract

**Objetivo:**

Este estudo analisou as tendências no uso de prótese dentária entre adultos e idosos e sua oferta no Sistema Único de Saúde (SUS) no Brasil, entre 2003 e 2023.

**Métodos:**

Estudo observacional ecológico quase-experimental de avaliação de impacto com um modelo *Difference-in-Differences* (DiD). Comparou-se a variação temporal entre municípios que implementaram Laboratórios de Prótese Dentária (LPD) (intervenção) e aqueles que não o fizeram (contrafactual). Foram utilizados dados de 43 municípios participantes de todos os inquéritos epidemiológicos SB Brasil (Saúde Bucal Brasil) de 2003, 2010 e 2023. As variáveis de desfecho foram o aumento do uso de próteses dentárias totais (PT), parciais removíveis (PPR) e fixas em adultos (35-44 anos) e idosos (65-74 anos) nestes municípios.

**Resultados:**

A presença de LPD entre 2003 e 2010 esteve associada ao aumento de 311,96% na oferta de próteses (p=0,032), mas foi estável entre 2010 e 2023. As mudanças de governo provavelmente impactaram a oferta de próteses nestes municípios. Ter LPD no período 2003-2010 e possuir Centro de Especialidades Odontológicas (CEO) entre 2010-2023 aumentou o uso de PT entre adultos em 6,94% (p=0,012) e 10,5% (p=0,032). Entre idosos, ter LPD aumentou o uso de PPR em 12,42% (p=0,002), e estar em município com CEO aumentou o uso de PT em 12,05% (p=0,05) entre 2003 e 2010.

**Conclusão:**

A oferta de LPD teve efeito residual. A menor perda dentária pode explicar a redução do uso de próteses em adultos e o aumento do uso de PPR em idosos. Sugerem-se análises futuras sobre cobertura, acesso e diferentes conjunturas.

## INTRODUÇÃO

A perda dentária continua sendo um problema de saúde pública de dimensão global, com impactos significativos sobre a qualidade de vida das pessoas^1^, ainda que os achados do SB Brasil 2023 apontem para a sua queda no Brasil entre adultos e idosos^2^. As consequências vão além do comprometimento das funções bucais e mastigatórias, estendendo-se a aspectos psicológicos e sociais, configurando a perda dentária como um desafio para os sistemas de saúde^3,4^.

A resposta social do Estado brasileiro foi a formulação de uma política de oferta de prótese dentária no Sistema Único de Saúde (SUS), em 2004, por meio da estratégia dos Laboratórios Regionais de Prótese Dentária, denominados no presente estudo como LPD, que realizam a etapa laboratorial da prótese, articulada com sua etapa clínica nos serviços de atenção primária à saúde ou especializada^5^. Em 2024, o Brasil entregou cerca de 1.237.151 próteses dentárias pelo SUS^6^, sendo que a maioria (51,5%) foram próteses totais (PT).

O acesso a próteses dentárias pelo sistema público de saúde, nos moldes adotados pelo Brasil, é incomum em outros países^7–9^. Quando ocorre, muitas vezes há compra desses serviços ao setor privado, com custos para os indivíduos^8,10^, apontados como um obstáculo ao acesso da população a esses serviços de saúde bucal^9,10^.

Os estudos que têm avaliado esse componente da Política Nacional de Saúde Bucal (PNSB) apontam dificuldades na oferta de especialistas e treinamentos das equipes; no planejamento e programação de atividades; levantamento das necessidades de prótese na população; monitoramento e análise dos indicadores de saúde bucal; além de baixo financiamento^11,12^, falta de apoio organizacional dos gestores^11,13^ e ausência de serviços odontológicos especializados^12,13^. Observou-se aumento da oferta, mas desigualdade na sua distribuição^14–16^, maior tempo de espera e necessidade de recorrer ao setor privado para obtenção de próteses dentárias^17^.

São escassas as pesquisas com enfoque avaliativo do impacto de políticas públicas, tendo sido recomendada sua incorporação^18^. Partiu-se da hipótese de que a ampliação da oferta de próteses dentárias no SUS aumentaria seu uso entre adultos e idosos nos municípios beneficiados em relação aos não beneficiados. Assim, este estudo avaliou as tendências do uso e o efeito da indução federal pelos LPD na oferta de próteses dentárias no SUS de 2003 a 2023 entre adultos e idosos brasileiros.

## MÉTODOS

Estudo observacional ecológico quase-experimental de avaliação de impacto com um modelo *Difference-in-Differences* (DiD)^18^, do tipo antes e depois com grupo contrafactual, com base nos inquéritos epidemiológicos SB Brasil 2003, 2010 e 2023, apenas com municípios participantes de todos os 3 levantamentos (n=43). O tamanho da amostra foi determinado pela disponibilidade dos dados provenientes dos inquéritos nacionais de saúde bucal realizados no período analisado, resultando na inclusão de 43 municípios com informações completas e comparáveis ao longo do tempo.

A análise foi conduzida em duas etapas. Na primeira, comparações descritivas de indicadores estratificados por grupos com e sem LPD foram realizadas, em alguns casos envolvendo um número reduzido de municípios e distribuições assimétricas. Por essa razão, optou-se por utilizar, de forma consistente em todas as comparações entre o início e o final do período, testes não paramétricos, com resultados por meio de medianas e intervalos interquartílicos. Na segunda etapa, realizou-se o ajuste de um modelo de regressão linear seguido de uma análise de resíduos.

Inicialmente, foram analisadas duas janelas temporais distintas (2003–2010 e 2010–2023), consideradas de forma independente. Em cada uma delas, compararam-se os percentuais de uso de PT, prótese parcial removível (PPR) e prótese fixa (PF) entre o início e o final do período, estratificados por municípios com e sem LPD e por duas faixas etárias (35–44 e 65–74 anos). Já a taxa de oferta de próteses por 100 mil habitantes, nos municípios selecionados, foi comparada entre o início e o final de cada período, estratificando por municípios com e sem LPD. Em todos os cenários, os indicadores foram descritos por meio da mediana e do intervalo interquartílico; e as comparações, realizadas com o teste de Wilcoxon.

As duas janelas temporais também foram analisadas na perspectiva de distintos períodos de governo, considerado neste estudo como um segmento específico da conjuntura, delimitado pelo intervalo temporal de atuação de uma equipe no poder responsável pela condução e execução das políticas públicas^19^.

Para a análise de impacto, utilizou-se o modelo DiD^18^ como estratégia para estimar o efeito da implementação dos LPD. Esse método compara a variação temporal no desfecho entre dois grupos: municípios com implementação do LPD (intervenção) e municípios sem LPD (controle). O desfecho considerado foi o percentual de uso de PT, PPR e PF antes e depois da política, em cada janela temporal (2003–2010 e 2010–2023), analisados separadamente para as duas faixas etárias (35–44 e 65–74 anos). O modelo linear estimado foi:


$$y_{it}=\beta_{0}+\beta_{1}LPD_{it}+\beta_{2}Ano_{it}+\beta_{3}LPD:Ano_{it}+\gamma x_{it}+\varepsilon_{it},$$

em que:

*y_it_* = proporção média de uso de prótese (total, removível ou fixa) do município *i* no ano *t*;

*LPD_it_* = indicador que assume valor 1 se o município *i* tiver LPD implementado no ano *t*, e 0, caso contrário;

*Ano_it_* = indicador que assume valor 1 para o ano final de cada janela, e 0 para o ano inicial;

*x_it_* = vetor de variáveis controle, incluindo condição de capital, presença de CEO e variação da taxa de analfabetismo entre os anos avaliados (valores menores deste indicador sugerem uma melhoria do município).

*ΰ*_0_ = intercepto do modelo;

*ΰ*_1_ = captura diferenças médias entre municípios com e sem LPD;

*ΰ*_2_ = efeito médio do tempo entre os dois períodos considerados;

*ΰ*_3_ = coeficiente que estima o efeito DiD da implementação dos LPD;

*γ* = vetor de parâmetros das variáveis de controle;

*ε_it_* = termo de erro.

A normalidade dos resíduos foi avaliada por meio do teste de Shapiro-Wilk. O nível de significância adotado para as análises foi de 5%, todas realizadas no software R (versão 4.4.3)^20^.

As variáveis de desfecho (percentual do uso de prótese dentária) foram construídas com base em dados agregados das % registradas por município em cada faixa etária, nos anos de 2003, 2010 e 2023, com base nos indivíduos examinados. Foi calculada a média em porcentagem de indivíduos que usavam algum tipo de prótese na arcada superior e inferior em cada grupo etário para cada município nos três inquéritos. Cabe destacar que os dados do SB Brasil 2003, 2010 e 2023 decorrem de delineamentos amostrais complexos e independentes. No presente estudo, os desfechos foram operacionalizados como proporções agregadas por município. Reconhece-se que seria possível calcular proporções ponderadas de acordo com os pesos individuais. Contudo, como os pesos amostrais individuais não estão disponíveis para o SB Brasil 2003, optou-se por uma abordagem ecológica e comparativa, sem ponderação individual.

Para a oferta do tipo de prótese dentária produzida, foram coletados dados, no Sistema de Informação Ambulatorial do SUS (SIA/SUS), sobre o quantitativo de próteses produzidas pelos LPD cadastrados dos 43 municípios incluídos neste estudo, nos meses de janeiro a dezembro de cada ano. A taxa anual de procedimentos de prótese dentária por 100 mil habitantes foi calculada dividindo o número total de próteses dentárias produzidas em cada município cadastrado pelo quantitativo da população do município na faixa etária acima dos 14 anos de idade, multiplicado por 100 mil. Para isso, o quantitativo populacional foi obtido utilizando os dados dos estudos "Estudo de estimativas populacionais por município, sexo e idade – 2000-2021" e "Projeção da População das unidades da federação por sexo, idade simples e grupos de idade: 2010-2060 (edição 2018)", disponíveis em: https://datasus.saude.gov.br/informacoes-de-saude-tabnet/^21^.

A nomenclatura e o código dos procedimentos ambulatoriais relativos à prótese dentária no SUS foram alterados em 2008. Assim, para os procedimentos realizados até 2007, foram coletadas informações dos seguintes procedimentos: 1008210 (prótese total mandibular), 1008211 (prótese total maxilar), 1008213 (prótese total mandibular), 1008214 (prótese total maxilar), 1008302 (próteses parciais remov. maxilar/mandibular), 1008303 (prótese parcial removível mandibular), 1008304 (prótese parcial removível maxilar), 1008406 (prótese parcial fixa por elemento), 1008407 (prótese adesiva metalocerâmica p/ elemento), 1008408 (prótese adesiva metaloplástica p/ elemento). A partir de 2008, foram coletadas as informações dos procedimentos ambulatoriais: prótese total mandibular (código SIGTAP 07.01.07.012-9), prótese total maxilar (código SIGTAP 07.01.07.013-7), prótese parcial mandibular removível (código SIGTAP 07.01.07.009-9), prótese parcial maxilar removível (código SIGTAP 07.01.07.010-2) e próteses coronárias/intrarradiculares fixas/adesivas (por elemento) (código SIGTAP 07.01.07.014-5)^6^.

Entre os 43 municípios analisados, 31 implementaram os LPD, sendo que a maior parte dos municípios com LPD iniciou sua implantação em 2009 (n=16, 37,2%) ou entre 2010 e 2014 (n=11, 25,6%). Os bancos de dados nacionais utilizados são produto da Pesquisa SB Brasil 2003, 2010 e 2023, e seus resultados são atualmente um banco público de acesso livre e gratuito. O relato do estudo seguiu as recomendações do checklist STROBE (*Strengthening the Reporting of Observational Studies in Epidemiology*). A tabela está disponível como Material Suplementar 1.

### Declaração de disponibilidade de dados:

Todo o conjunto de dados que dá suporte aos resultados deste estudo foi publicado no próprio artigo

## RESULTADOS

No período 2003-2010, verificou-se aumento estatisticamente significativo na oferta de próteses dentárias nos municípios com LPD. A mediana passou de 0,4 para 306,3 próteses/100 mil habitantes (p=0,0074). Nos municípios sem LPD, não houve alteração significativa. Já entre 2010 e 2023, não se observou diferença na oferta em nenhum dos grupos (Tabela 1). Na análise de DiD, a presença de LPD em 2003-2010 esteve associada a um aumento de 311,96% na oferta de próteses (p=0,032) (Tabela 2).

**Tabela 1:** Mediana e quartis inferior e superior das variáveis de desfecho "oferta de prótese dentária no SUS" (população total) e "uso de próteses dentárias por tipo" (total, parcial removível ou fixa unitária) entre adultos e idosos nos anos de 2003–2010 e 2010–2023, p-valor e tendência em municípios com e sem laboratórios de prótese dentária no Brasil (n=43).

Período SB Brasil e Presença LPD e variável de desfecho		2003–2010			2010–2023		
		2003	2010	p-valor e tendência	2010	2023	p-valor e tendência
		mediana (IIQ)	mediana (IIQ)		mediana (IIQ)	mediana (IIQ)	
Taxa por 100 mil de oferta de prótese							
	Sem LPD	0	0,3	0,0884	0	0	0,2733
		(0–37,4)	(0–92,7)	(Estável)	(0–9,7)	(0–5,3)	(Estável)
	Com LPD	0,4	306,3	**0,0074**	93,3	80	0,1447
		(0–36,7)	(53,1–410,9)	(Aumento)	(0,3–376,2)	(11,0–175,5)	(Estável)
% de uso de PT aos 35–44 anos							
	Sem LPD	18,2	7,8	**0,0014**	12,1	4,3	0,1441
		(13,3–24,1)	(5,3–16,0)	(Diminuição)	(7,7–23,1)	(1,9–11,8)	(Estável)
	Com LPD	21,1	7,9	**0,0019**	7,7	2,2	**0,0012**
		(14,7–27,5)	(5,9–8,9)	(Diminuição)	(5,5–8,7)	(0,9–3,5)	(Diminuição)
% de uso de PPR aos 35–44 anos							
	Sem LPD	30,2	23,1	**0,0345**	24,5	15,5	0,1614
		(20,3–33,3)	(18,1–28,8)	(Diminuição)	(21,6–28,5)	(9,1–23,2)	(Estável)
	Com LPD	22,6	16,3	0,2343	19,4	7,5	**<0,0001**
		(21,5–28,8)	(12,4–33,1)	(Estável)	(14,9–31,8)	(4,9–12,9)	(Diminuição)
% de uso de PF aos 35–44 anos							
	Sem LPD	10,3	3,8	**0,0413**	4,1	4	1,0000
		(5,1–12,3)	(1,1–7,3)	(Diminuição)	(1,2–8,0)	(3–4,6)	(Estável)
	Com LPD	7	2,9	**0,0341**	3	2,2	0,5663
		(3,5 - 11,6)	(1–4,2)	(Diminuição)	(1,1–6,4)	(1,1–3,5)	(Estável)
% de uso de PT aos 65–74 anos							
	Sem LPD	55,2	59,6	0,8291	61,5	62,5	0,9528
		(50–69,0)	(54,5–63,4)	(Estável)	(57,3–64,1)	(43,0–76,90	(Estável)
	Com LPD	60	61,1	0,554	59,2	51	**0,0014**
		(51,7–64,3)	(55,6–65,3)	(Estável)	(54,5–64,6)	(46,7–59,4)	(Estável)
% de uso de PPR aos 65–74 anos							
	Sem LPD	14,3	2,6	0,1208	12,5	25	**0,0180**
		(9,1–16,7)	(0,9–12,8)	(Estável)	(5,7–13,6)	(18,4–28,6)	(Aumento)
	Com LPD	21,4	2,4	**0,0231**	2,4	23,9	**0,0026**
		(17,4–28,6)	(1,2–6,7)	(Diminuição)	(1,2–5,5)	(20,0–26,7)	(Aumento)
% de uso de PF aos 65–74 anos							
	Sem LPD	10,4	9,5	0,8785	10,5	2	0,1088
		(6,8–15,4)	(6,2–13,1)	(Estável)	(6,5–12,5)	(1,7–2,70)	(Estável)
	Com LPD	10	7,5	0,7532	7,8	4,8	**<0,0001**
		(5,3–12,3)	(4,8–12,6)	(Estável)	5,3–13,1	(3,7–7,3)	(Diminuição)

LPD: laboratórios de prótese dentária; PT: prótese total; PPR: prótese parcial removível; PF: prótese fixa. Os valores de p em negrito confirmam a significância estatística da associação adotada para as análises, que foi de 5% (<0,05).

Fonte: Dados do SB Brasil 2003, 2010 e 2023.

**Tabela 2:** Efeito da presença de laboratórios de prótese dentária na variação da oferta (população total) e do uso de próteses dentárias entre adultos (35–44 anos) nos períodos 2003–2010 e 2010–2023 em municípios selecionados (n=43) com base no modelo DiD.

Variável	Efeito estimado	Erro padrão	Estatística	Valor p	Variável	Efeito estimado	Erro padrão	Estatística	Valor p
2003–2010					2010–2023				
Taxa de oferta de prótese por 100 mil hab.					Taxa de oferta de prótese por 100 mil hab.				
Intercepto	51.691	160.466	0.322	0.748	Intercepto	255.359	526.804	0.485	0.630
Taxa Analf 2010–2003	-1.259	11.108	-0.113	0.910	Taxa Analf 2023–2010	6.963	23.543	0.296	0.769
CEO 2003–2010 (Sim)	-12.391	133.023	-0.093	0.926	CEO (Sim)	-73.057	475.186	-0.154	0.878
Capital	-2.939	73.605	-0.040	0.968	Capital	**-236.645**	**111.782**	**-2.117**	**0.039**
Ano (2010)	41.954	131.249	0.320	0.750	Ano (2023)	-142.809	445.413	-0.321	0.750
LPD (Sim)	74.735	101.738	0.735	0.465	LPD (Sim)	305.243	522.931	0.584	0.562
Ano (2010): LPD (Sim)	**311.960**	**143.016**	**2.181**	**0.032**	Ano (2023): LPD (Sim)	-72.367	456.170	-0.159	0.875
**Uso de prótese fixa**					**Uso de prótese fixa**				
Intercepto	14.501	4.820	3.008	0.004	Intercepto	10.009	1.749	5.724	< 0.001
Taxa Analf 2010–2003	**-0.546**	**0.208**	**-2.626**	**0.011**	Taxa Analf 2023–2010	0.120	0.128	0.936	0.354
CEO 2003–2010 (Sim)	-2.273	4.754	-0.478	0.634	CEO (Sim)	-3.464	2.363	-1.466	0.149
Capital	**-2.730**	**1.268**	**-2.153**	**0.035**	Capital	**-5.219**	**1.004**	**-5.199**	**<0.001**
Ano (2010)	-6.246	4.715	-1.325	0.190	Ano (2023)	-1.556	2.009	-0.774	0.442
LPD (Sim)	-2.595	1.608	-1.614	0.112	LPD (Sim)	-1.226	1.634	-0.750	0.457
Ano (2010): LPD (Sim)	0.340	2.293	0.148	0.883	Ano (2023): LPD (Sim)	0.183	2.165	0.084	0.933
**Uso de prótese parcial removível**					**Uso de prótese parcial removível**				
Intercepto	19.658	5.341	3.680	<0.001	Intercepto	26.508	4.506	5.883	<0.001
Taxa Analf 2010–2003	-0.117	0.374	-0.313	0.755	Taxa Analf 2023-2010	-0.004	0.315	-0.013	0.990
CEO 2003–2010 (Sim)	6.412	4.421	1.450	0.151	CEO (Sim)	2.523	4.772	0.529	0.599
Capital	1.566	2.505	0.625	0.534	Capital	**-10.316**	**2.894**	**-3.565**	**0.001**
Ano (2010)	1.841	4.309	0.427	0.670	Ano (2023)	-5.100	4.875	-1.046	0.299
LPD (Sim)	-2.173	3.342	-0.650	0.518	LPD (Sim)	4.233	4.546	0.931	0.355
Ano (2010): LPD (Sim)	2.043	4.791	0.426	0.671	Ano (2023): LPD (Sim)	-5.892	5.503	-1.071	0.288
**Uso de prótese total**					**Uso de prótese total**				
Intercepto	17.653	4.473	3.946	<0.001	Intercepto	15.028	3.813	3.942	<0.001
Taxa Analf 2010–2003	0.219	0.292	0.751	0.455	Taxa Analf 2023-2010	0.449	0.313	1.437	0.156
CEO 2003–2010 (Sim)	5.834	3.799	1.536	0.129	CEO (Sim)	**10.500**	**4.774**	**2.199**	**0.032**
Capital	**-9.670**	**2.077**	**-4.655**	**<0.001**	Capital	**-12.816**	**2.951**	**-4.343**	**<0.010**
Ano (2010)	-1.668	3.772	-0.442	0.660	Ano (2023)	1.727	4.796	0.360	0.720
LPD (Sim)	**6.936**	**2.682**	**2.586**	**0.012**	LPD (Sim)	5.201	3.848	1.352	0.182
Ano (2010): LPD (Sim)	-6.132	3.905	-1.570	0.121	Ano (2023): LPD (Sim)	-4.522	5.229	-0.865	0.391

LPD: laboratórios de prótese dentária. Os valores de p em negrito confirmam a significância estatística da associação adotada para as análises, que foi de 5% (<0,05).

Fonte: Dados do SB Brasil 2003, 2010 e 2023.

Entre adultos de 35-44 anos, o uso de próteses reduziu de forma significativa ou apresentou estabilidade em todos os tipos entre 2003 e 2010, tanto nos municípios com LPD quanto nos sem LPD. Nos municípios sem LPD, a redução foi significativa entre 2003 e 2010, passando de 30,2% para 23,1% (p=0,0345). Para a PF, houve redução nos grupos tanto com quanto sem LPD (p=0,04 e p=0,03, respectivamente). Para a PT, a mediana caiu de 18,2% para 7,8% (p=0,0014) nos municípios sem LPD e de 21,1% para 7,9% (p=0,0019) nos com LPD (Tabela 1). Já no período 2010-2023, entre os adultos a redução foi novamente significativa para PT e PPR no grupo com LPD, chegando a apenas 2,2% de uso de PT entre adultos em 2023 e apresentando estabilidade nos grupos de municípios sem LPD (Tabela 1, Figura 1).

**Figura 1:** Percentual médio e intervalo de confiança de 95% para o uso de próteses dentárias, segundo tipo de prótese e faixa etária (35–44 e 65–74 anos), em municípios com e sem participação na Política de Laboratórios de Prótese Dentária. A primeira linha contém a comparação do período 2003–2010 e a segunda, do período de 2010–2023.

Na análise DiD para o período 2003-2010, chama a atenção que a redução da taxa de analfabetismo esteve associada ao menor uso de prótese fixa em adultos em -0,546% (p=0,011). Já estar em uma capital reduziu o uso de PF e PT em -2,73% (p=0,035) e -9,67% (<0,001), respectivamente. Além disso, os municípios com LPD apresentaram um aumento no uso de PT de 6,94% (p=0,012). No período 2010-2023, morar em uma capital foi associado à redução do uso de todos os tipos de próteses entre adultos. Por outro lado, aumentou-se o uso de PT entre adultos em 10,5% (p=0,032) em municípios com CEO (Tabela 2).

Entre idosos de 65 a 74 anos, no período de 2003-2010, houve uma estabilidade em quase todos os tipos, com exceção da diminuição do uso de PPR em municípios com LPD (p=0,0231). Por sua vez, no período de 2010-2023, chama a atenção o aumento significativo do uso de PPR em municípios com e sem LPD. Para o uso de PT, houve estabilidade nos municípios com (p=0,0014) e sem LPD (p=0,9528), sem significância. Para o período 2003-2010, o efeito estimado foi de 4,5% no aumento do uso de PT nos municípios com LPD, mas não significativo (Tabela 3). Ainda entre os idosos, nas capitais houve associação com maior redução no uso de PPR em 13,5% (p<0,001) entre 2003 e 2010, e redução de 15,65% (p<0,001) entre 2010 e 2023. Por outro lado, entre 2003 e 2010, em municípios com LPD, houve associação com maior uso de PPR em 12,42% (p=0,002). Já no período 2010-2023, em municípios com CEO, houve associação com maior uso de PT neste grupo em 12,05% (p=0,049).

**Tabela 3:** Efeito da presença de laboratórios de prótese dentária na variação do uso de próteses dentárias entre idosos (65–74 anos) nos períodos 2003–2010 e 2010–2023 em municípios selecionados (n=43) com base no modelo DiD.

Variável	Efeito estimado	std.error	statistic	Valor p	Variável	Efeito estimado	std.error	statistic	Valor p
2003–2010					2010–2023				
% de uso de prótese fixa					Uso de prótese fixa				
Intercepto	10.814	5.458	1.981	0.054	Intercepto	13.153	4.221	3.116	0.003
Taxa Analf 2010–2003	-0.036	0.324	-0.110	0.913	Taxa Analf 2023–2010	0.454	0.271	1.673	0.100
CEO (Sim)	1.086	4.576	0.237	0.813	CEO (Sim)	3.714	5.274	0.704	0.484
Capital	-0.953	2.491	-0.383	0.704	Capital	**-6.195**	**2.020**	**-3.066**	**0.003**
Ano (2010)	0.801	4.411	0.182	0.857	Ano (2023)	-4.388	4.781	-0.918	0.363
LPD (Sim)	-1.634	3.382	-0.483	0.631	LPD (Sim)	3.608	3.925	0.919	0.362
Ano (2010): LPD (Sim)	0.361	4.195	0.086	0.932	Ano (2023): LPD (Sim)	-0.997	5.020	-0.199	0.843
**Variação % de uso de prótese parcial removível**					**Uso de prótese parcial removível**				
Intercepto	18.534	6.354	2.917	0.005	Intercepto	18.873	3.979	4.743	<0.001
Taxa Analf 2010–2003	-0.483	0.445	-1.085	0.283	Taxa Analf 2023–2010	0.095	0.255	0.374	0.710
CEO 2003–2010 (Sim)	4.333	5.368	0.807	0.423	CEO (Sim)	-6.195	3.978	-1.557	0.124
Capital	**-13.506**	**3.009**	**-4.488**	**< 0.001**	Capital	**-15.650**	**2.566**	**-6.100**	**<0.001**
Ano (2010)	-0.461	5.386	-0.086	0.932	Ano (2023)	**14.987**	**4.365**	**3.433**	**<0.001**
LPD (Sim)	**12.426**	**3.865**	**3.215**	**0.002**	LPD (Sim)	3.425	4.126	0.830	0.409
Ano (2010): LPD (Sim)	-9.798	5.365	-1.826	0.073	Ano (2023): LPD (Sim)	-1.193	4.966	-0.240	0.811
**Variação % de uso de prótese total**					**Uso de prótese total**				
Intercepto	65.163	8.938	7.290	< 0.001	Intercepto	55.057	6.306	8.730	<0.001
Taxa Analf 2010–2003	-0.547	0.619	-0.884	0.380	Taxa Analf 2023–2010	0.542	0.409	1.324	0.189
CEO 2003–2010 (Sim)	-3.016	7.409	-0.407	0.685	CEO (Sim)	**12.046**	**6.040**	**1.994**	**0.049**
Capital	-1.607	4.100	-0.392	0.696	Capital	-6.849	3.735	-1.834	0.071
Ano (2010)	-2.607	7.311	-0.357	0.722	Ano (2023)	4.215	6.717	0.628	0.532
LPD (Sim)	-6.184	5.667	-1.091	0.278	LPD (Sim)	10.350	6.278	1.649	0.103
Ano (2010): LPD (Sim)	4.525	7.966	0.568	0.572	Ano (2023): LPD (Sim)	-11.593	7.604	-1.525	0.131

LPD: laboratórios de prótese dentária. Os valores de p em negrito confirmam a significância estatística da associação adotada para as análises, que foi de 5% (<0,05).

Fonte: Dados do SB Brasil 2003, 2010 e 2023.

No que se refere às tendências, chama a atenção a diminuição ou estabilidade do uso de próteses entre adultos. Já entre os idosos, houve estabilidade ou diminuição na porcentagem de uso de próteses, com exceção do aumento de uso de PPR no segundo período neste grupo em municípios com e sem LPD (Tabela 1).

## DISCUSSÃO

Os achados do presente estudo indicam que a taxa de oferta de prótese por 100 mil habitantes aumentou entre 2003 e 2010, mas manteve-se estável entre 2010 e 2023. As mudanças de conjuntura, mais favoráveis no primeiro período – governos Lula I e II (2003–2010) – e restritiva ao final do segundo período (2010–2023), provavelmente impactaram na oferta de próteses nos municípios analisados. A PNSB, também chamada de Brasil Sorridente, foi uma das quatro políticas prioritárias na agenda federal a partir de 2003, e a oferta pública de próteses dentárias passou a ter uma indução federal regular com a criação dos LPD^16,22^. Paim^23^ aponta que forças políticas e sociais passaram a ocupar espaços nesse período, com fortalecimento das políticas sociais na agenda política.

Houve um crescimento dos repasses financeiros para a saúde bucal realizados pela União até 2013 e manutenção de valores entre 2013 e 2016; contudo, houve redução a partir de 2017 e grande queda em 2018^24^. Isso converge com a elevação da taxa de oferta de próteses por 100 mil habitantes no primeiro período, mas as instabilidades nos governos após 2015 reduziram a capacidade de indução federal, somadas às dificuldades no nível local. A crise financeira vivenciada pelo Brasil em 2008 não apresentou reflexos diretos no financiamento para a saúde bucal, pela rápida recuperação pelo governo federal. Entretanto, entre 2011 e 2016, o cenário econômico e político enfrentou medidas de austeridade, assim, o espaço para consolidação de um projeto nacional de expansão das políticas sociais foi restringido^24^.

O período de 2016–2022 foi marcado pelo aprofundamento de medidas de austeridade fiscal no âmbito nacional, além de contrarreformas e crises políticas no Estado brasileiro^25,26^. A política de oferta de prótese dentária ampliou a capacidade instalada do SUS, ainda que tal expansão não tenha se traduzido proporcionalmente em maior utilização pela população, refletida na maior proporção de uso de próteses. Cabe destacar que a implantação dos LPD nos municípios concentrou-se entre 2009 e 2010, com crescimento substancial de LPD no período e maior registro em 2023 (n=3.737) – um aumento de aproximadamente 42 vezes em relação ao ano de 2005, sendo que a maioria deles é de natureza jurídica privada^6,21^. Além disso, estudo anterior revelou que, nos municípios com LPD, a mediana passou de 36,7 próteses/100 mil habitantes em 2003 para 376,2 em 2010 (p=0,031)^27^. Esses estudos sugerem que a implantação dos LPD atuou como indutor da produção de próteses no SUS, permitindo maior disponibilidade desse tipo de reabilitação^27^.

O impacto não observado na maior utilização de próteses dentárias nos municípios com LPD requer análise cuidadosa. Primeiro, chama a atenção, na análise DiD, que o período de 20 anos entre os 3 inquéritos exerceu grande influência na redução do uso de prótese entre adultos, ainda que em menor proporção entre idosos^2,3^. Além disso, ainda que os municípios com LPD tenham se ampliado nesse período, a oferta de próteses apresentou variações abaixo das faixas de financiamento induzidas pelo Ministério da Saúde^6^. As razões podem estar relacionadas às dificuldades nos processos licitatórios, com descontinuidade dos contratos com os LPD privados e baixo número de laboratórios privados interessados em participar de licitações. Por fim, municípios com maior porte populacional, como as capitais, que formam a maioria da presente amostra, apresentam baixa concentração de LPD por habitante^15,21^. Em síntese, na amostra do presente estudo estão todas as capitais, as quais, mesmo com serviço de prótese dentária, sua baixa cobertura pode resultar em baixo acesso e, logo, não impactar o indicador de uso de próteses.

Sobre modelos de licitação competitiva, estes podem resultar em negociações complexas, prazos alongados, aumento de custos e queda na qualidade dos serviços, sugerindo a necessidade de monitoramento rigoroso^28^.

Estudo apontou que municípios com maior presença de CEO e ESB (Equipe de Saúde Bucal) apresentaram maior oferta^29^, mas, ainda assim, a quantidade de próteses entregues ficou abaixo do esperado, e a tendência manteve-se estável. Além disso, o estudo também revelou que persistem desigualdades raciais, pois, mesmo onde há maior oferta, a população negra recebe proporcionalmente menos próteses do que a população branca, com uma diferença de 7,7% em desfavor dos negros. Essa desigualdade é mais acentuada em cidades com mais ESB e/ou maior proporção de população negra^29^.

Estudos nacionais indicam que a necessidade de prótese dentária entre idosos brasileiros é alta, mas tem se reduzido ao longo dos últimos inquéritos^2,3,29,30^, consequentemente levando a menor prevalência do uso de próteses^29,30^. Contudo, vale destacar que essa necessidade é especialmente maior em populações de baixa renda, cor da pele não branca e menor escolaridade^29,30^. Indivíduos com menor escolaridade e renda apresentam maior necessidade e menor uso de próteses, refletindo desigualdades históricas no acesso à saúde bucal^4,30,31^. Estudos longitudinais mostram estabilidade na prevalência de uso de prótese entre idosos em São Paulo entre 2000 e 2010, sem redução significativa no período^32^. No caso dos inquéritos epidemiológicos, observou-se uma redução da necessidade entre adultos de 75,9% em 2010 para 53,57% em 2023. Entre os idosos, 96% necessitavam de próteses de qualquer tipo em 2010 e, em 2023, este percentual reduziu para 75,2%^2,3^.

A diminuição do uso de próteses é consequência das mudanças no perfil de saúde bucal da população, com redução da perda dentária em algumas faixas etárias devido à ampliação de políticas de prevenção e promoção de saúde bucal^33^. O aumento de uso de PPR é, provavelmente, também produto da menor perda dental observada na série histórica no Brasil. Em países europeus, cerca de metade dos adultos possui algum tipo de prótese dentária, com tendência de redução do edentulismo e aumento de próteses fixas e implantes^34^. Nesse sentido, a incorporação de próteses fixas e sobre implantes pode ser um recurso importante para a reabilitação oral, já consolidada em outros países^34^, com o avanço das tecnologias digitais^34^.

Apesar da ampla adoção de instrumentos padronizados como o *Oral Health-Related Quality of Life* (OHRQoL)^35^, não foram encontrados estudos com o uso de métodos de avaliação de impacto robustos, como DiD ou Séries Temporais Interrompidas (ITS), para políticas públicas ou programas de saúde bucal. Revisões sistemáticas e de escopo apontam que a maioria das avaliações foca em desfechos clínicos ou autorreferidos, com foco em grupos escolares ou crianças, sem explorar métodos de inferência causal para políticas em larga escala^36,37^. Nesse sentido, o presente estudo propõe desfechos relacionados ao objetivo declarado da política e sugere incorporar medidas específicas baseadas em modelos teóricos. A adoção de métodos de avaliação de impacto como DiD e ITS é recomendada para fortalecer a evidência sobre a efetividade de políticas de saúde bucal, especialmente em contextos mais amplos, com uso dos sistemas de informação já disponíveis^36,19^.

O presente estudo apresenta limitações relacionadas à utilização de dados secundários, agregados por município. Além disso, os dados do SB Brasil 2003, 2010 e 2023 decorrem de delineamentos amostrais complexos e independentes. No entanto, no presente estudo, os desfechos foram operacionalizados como proporções agregadas por município, e o objetivo analítico foi estimar variações temporais relativas e diferenças entre grupos de municípios, e não produzir estimativas populacionais nacionais. Reconhece-se que seria possível calcular proporções ponderadas com base nos pesos individuais. Contudo, os pesos amostrais não estão disponíveis para o SB Brasil 2003, o que inviabilizaria sua incorporação de forma consistente ao longo de toda a série histórica analisada. Diante disso, optou-se por uma abordagem ecológica e comparativa, reconhecendo essa decisão como uma limitação metodológica do estudo. Outra limitação foi que a amostra final compreendeu apenas municípios presentes nos 3 inquéritos (n=43), o que resultou em maior representação de capitais e municípios de grande porte. Para mitigar possíveis desequilíbrios regionais, incluímos uma variável indicador "ser capital" nos modelos ajustados.

Sugere-se que estudos de avaliação de impacto incorporem dados individualizados com foco em posição social (renda e escolaridade), tipo de serviço utilizado, necessidade de prótese e percepções sobre saúde bucal. Recomenda-se, também, que novos estudos investiguem as diferenças de oferta de prótese por porte populacional e rede organizativa, bem como análises do período 2003–2023. Considerando a abrangência nacional dos dados e o caráter universal do SUS, os achados apresentam potencial de generalização para contextos semelhantes.

Por fim, a perda dentária, entendida como expressão de iniquidades sociais e do acesso precário à atenção odontológica ao longo da vida, ainda se constitui importante problema de saúde pública que fundamentou a política de próteses dentárias no SUS^5,31^.

## Material suplementar

Material suplementar 1
